# Deep learning model to discriminate diverse infection types based on pairwise analysis of host gene expression

**DOI:** 10.1016/j.isci.2024.109908

**Published:** 2024-05-07

**Authors:** Jize Xie, Xubin Zheng, Jianlong Yan, Qizhi Li, Nana Jin, Shuojia Wang, Pengfei Zhao, Shuai Li, Wanfu Ding, Lixin Cheng, Qingshan Geng

**Affiliations:** 1 Guangdong Provincial Clinical Research Center for Geriatrics, Shenzhen People’s Hospital (First Affiliated Hospital of Southern University of Science and Technology, Second Clinical Medicine College of Jinan University), Shenzhen 518020, China; 2 John Hopcroft Center for Computer Science, Shanghai Jiao Tong University, Shanghai, China; 3 Great Bay University, Dongguan, China; 4 Health Data Science Center, Shenzhen People’s Hospital, Shenzhen 518020, China

**Keywords:** Pathophysiology, Clinical microbiology, Medical informatics, Biocomputational method, Neural networks

## Abstract

Accurate detection of pathogens, particularly distinguishing between Gram-positive and Gram-negative bacteria, could improve disease treatment. Host gene expression can capture the immune system’s response to infections caused by various pathogens. Here, we present a deep neural network model, bvnGPS2, which incorporates the attention mechanism based on a large-scale integrated host transcriptome dataset to precisely identify Gram-positive and Gram-negative bacterial infections as well as viral infections. We performed analysis of 4,949 blood samples across 40 cohorts from 10 countries using our previously designed omics data integration method, iPAGE, to select discriminant gene pairs and train the bvnGPS2. The performance of the model was evaluated on six independent cohorts comprising 374 samples. Overall, our deep neural network model shows robust capability to accurately identify specific infections, paving the way for precise medicine strategies in infection treatment and potentially also for identifying subtypes of other diseases.

## Highlights

• bvnGPS2 machine learning model allows to distinguish between four infection types
• The model is based on 4,949 blood samples across 40 cohorts
• bvnGPS2 shows superiority over traditional machine learning methods
• bvnGPS2 has the potential to assist the diagnosis of infections in clinical practice

## Graphical abstract

**Figure:**
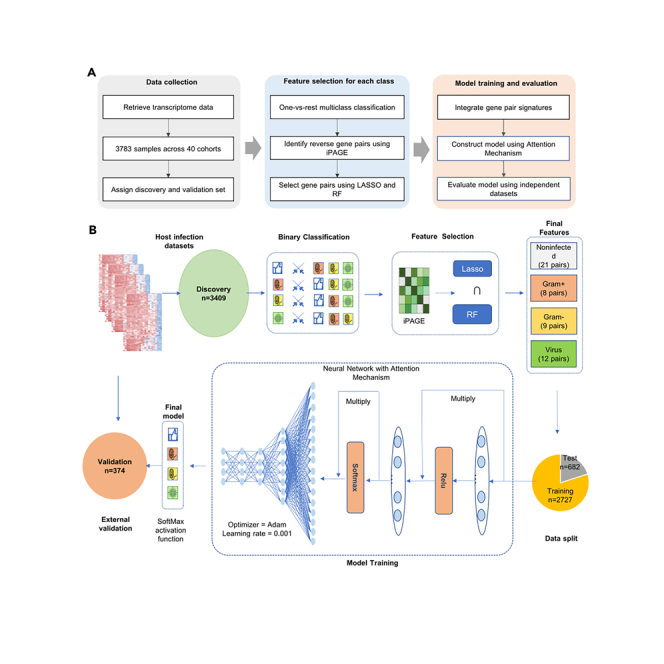

## Introduction

Infectious diseases occur when the immune system reacts to the invasion of pathogens, such as viruses, bacteria, or fungi. The emergence of drug-resistant bacteria and the global impact of fatal viruses, such as SARS-CoV-2 causing the COVID-19 pandemic, have intensified the public health challenge.^1^ As the treatment strategies for different bacterial, viral, and non-infectious inflammation are distinct, accurate diagnosis of pathogens is crucial for reducing mortality and improving public health in the long term.^2^^,^^3^ The immune system responds specifically to combat pathogens, leading to differential gene expression in the host. Gene expression, therefore, holds the potential as blood-based biomarkers for infection detection,^2^^,^^4^^,^^5^^,^^6^ especially for the early detection of acute infection with the help of polymerase chain reaction (PCR) technology.^7^^,^^8^^,^^9^

Accumulating computational models have been developed to leverage the host immune response to predict the presence and type of infections.^2^^,^^10^^,^^11^ However, most of these works are not applicable for clinical practice either because of the small sample size^10^^,^^12^ or the limited infection categories ^[^^2^^,^^13^^,^^14^^,^^15^^,^^16^^].^ To integrate multiple cohorts suffering from batch effect, previous studies usually utilized data normalization or network analysis,^2^^,^^10^^,^^17^^,^^18^^,^^19^^,^^20^ which would be verbose for clinical practice. For instance, some model requires a dataset including non-infected samples as a reference before diagnosing unknown patients.^10^

In our earlier work, we proposed a method named bvnGPS to discriminate bacterial infection, viral infection, and non-infection, based on integrated host transcriptome data and pretrained neural networks.^12^ This method used a dataset that integrated various host transcriptomic data and extracted the common features across different cohorts by comparing the relative expression changes of gene pairs.^12^ Benefited from the effectiveness of individualized Pairwise Analysis of Gene Expression (iPAGE)^11^^,^^21^^,^^22^^,^^23^ and deep neural network model, bvnGPS achieved a high performance in determining distinct infection types.

Based on the previous work, it is important to develop a model to discriminate Gram-positive bacterial infection and Gram-negative bacterial infection due to three reasons. First, it can suggest the appropriate antibiotic treatments, as different types of antibiotics are effective against different types of bacteria. Second, it is useful when monitoring the spread and resistance of bacterial infections, as Gram-negative bacteria are more likely to develop resistance to antibiotics than Gram-positive bacteria. Third, it helps to understand the pathogenesis and immune response of bacterial infections, as Gram-positive and Gram-negative bacteria have different cell wall structures and release different toxins.^24^^,^^25^^,^^26^

Here, we aimed to expand the discrimination scope of bvnGPS to four infection categories: Gram-positive bacterial infection, Gram-negative bacterial infection, viral infection, and non-infection. To improve the performance of multi-label classification, we substantially enlarged the sample size of each infection type and updated the prediction model with the attention mechanism.

**Figure 1:**
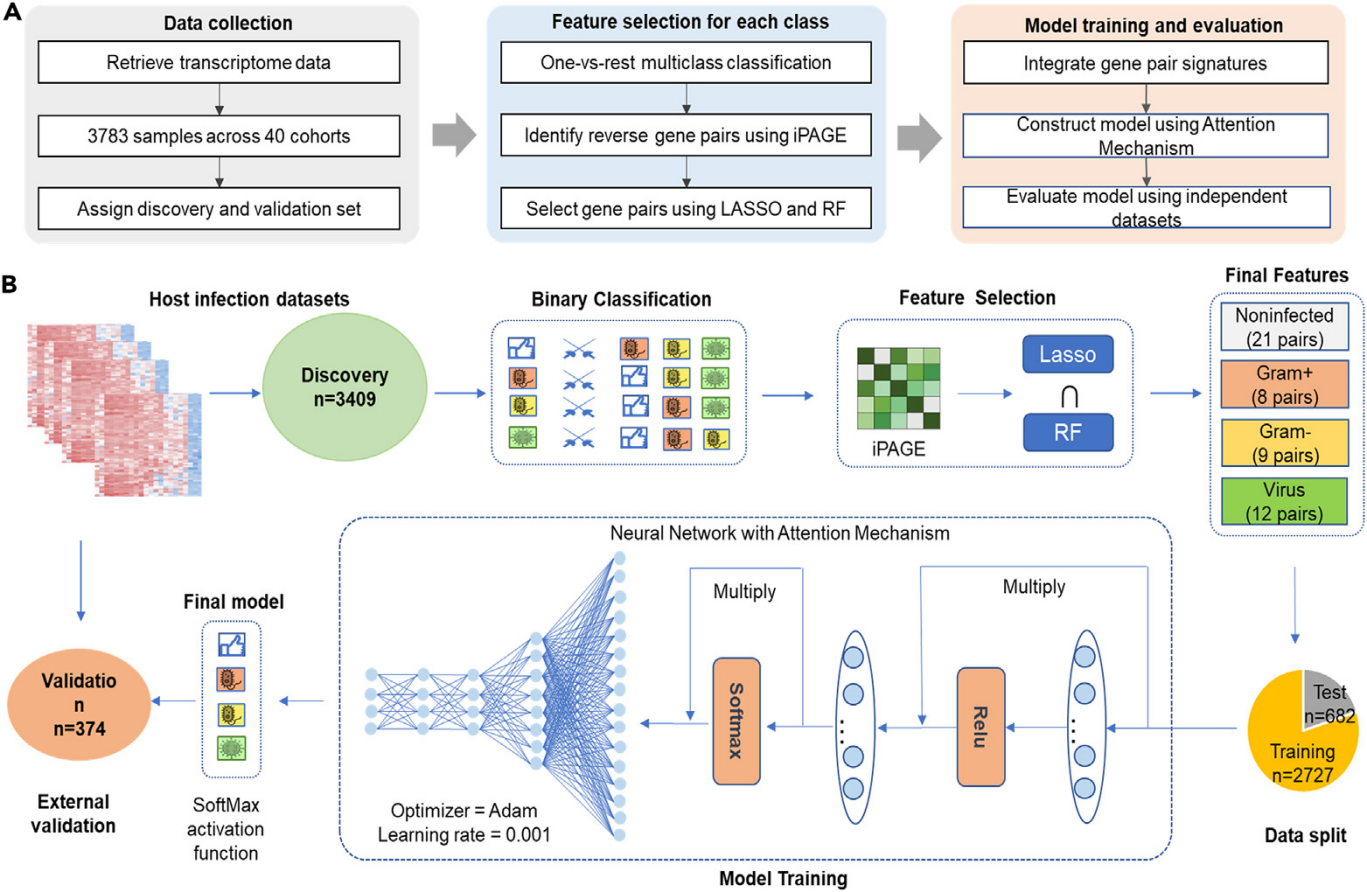
Workflow of this study (A) The workflow of this study from data collection, feature selection to model training and evaluation. (B) The detailed framework of bvnGPS2: the host infection datasets were split into a discovery set and validation set. Gene pair signatures for non-infection, Gram+ bacterial, Gram-bacterial, and viral infections were extracted by iPAGE. After that, gene pairs selected by LASSO regression and RF were used as final features for the deep neural network with an attention mechanism. The discovery data were further split into a training set for network training and tuning, and a test set for internal evaluation. Then, bvnGPS2 with two attention layers and five fully connected layers was trained. Finally, the external validation was performed for bvnGPS2.

In this work, we developed a robust multi-class decision model, bvnGPS2, that could differentiate between infections caused by Gram-positive bacteria, Gram-negative bacteria, virus (mainly focusing on RNA virus) and non-infection, based on the generalizable gene expression pattern in human whole blood.^11^ First, we compiled a comprehensive dataset of 4,949 samples from 40 cohorts and applied iPAGE to integrate multiple infectious cohorts and reduce batch effects.^11^^,^^27^^,^^28^ Subsequently, we constructed the bvnGPS2 model to detect infections based on the attention mechanism using 50 discriminant gene pairs (DGPs) identified by LASSO and Random Forest. Finally, we evaluated the performance of bvnGPS2 on six independent validation cohorts (Figure 1). Our results demonstrated the superiority of bvnGPS2 over traditional machine learning methods, including Support Vector Machine, Random Forest, and Gradient Boosting Decision Tree. Notably, the relative expression used in bvnGPS2 is comparable between samples in different cohorts, highlighting the potential applicability of our approach in clinical settings and for determining subtypes of other diseases.

## Results

### Host transcriptome data integration

**Figure 2:**
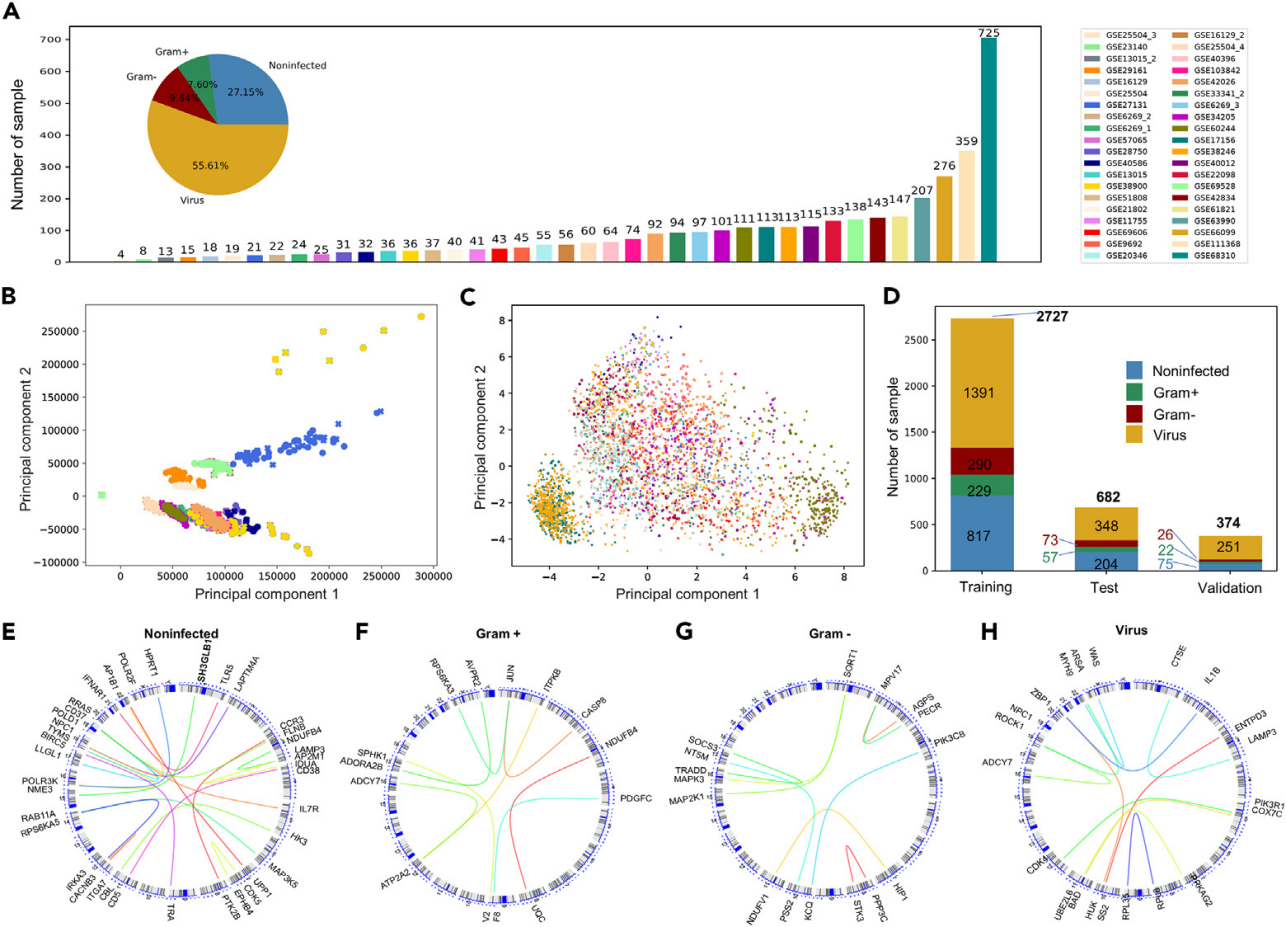
Transcriptome data integration (A) Datasets overview. Bar chart showing the number of samples in each cohort. Pie chart showing the distribution of samples among the four infection types. (B and C) Principal component analysis showing the clusters of samples based on their similarity before (B) and after (C) batch effect correction. Dots represent samples and colors represent different cohorts. (D) The number of samples in the training set, test set, and validation set. (E–H) The genomic locations of the selected gene pairs for non-infection (E), Gram+ bacterial infection (F), Gram-bacterial infection (G), and viral infection (H).

We collected all the cohorts about infection in GEO database and integrated these cohorts, resulting in 4,949 samples in 40 host transcriptome cohorts (Figure 2A). After filtering out samples with coinfection, unknown infection, and missing information, 3,783 samples were remained in this study (Table 1; Figure S1).

**Table 1:** Transcriptome datasets used in this work

Data Accession	Organs	Cohort Details	Pathogen	Platform	Total	Non-infected	Gram +	Gram -	Virus
**Discovery set**									

GSE6269	Whole blood	Children	Influenza A, Influenza B, S. pneumoniae, S. aureus, MSSA, MRSA	GPL2507	24	0	16	0	8
GSE6269	Whole blood	Both	Influenza A, Influenza B, S. pneumoniae, S. aureus, MSSA, MRSA	GPL570	22	0	12	0	10
GSE6269	Whole blood	Both	S. aureus, MSSA, E. coli, S. pneumoniae, Influenza A	GPL96	97	6	44	29	18
GSE11755	Whole blood	Children		GPL570	41	10	0	31	0
GSE13015	Whole blood	Adult	E.coli, B.pseudomallei	GPL6947	39	5	0	8	0
GSE16129	Whole blood	Children	Bacteremia, Osteomyelitis	GPL6106	18	9	9	0	0
GSE16129	Whole blood	Both	Bacteremia, Osteomyelitis	GPL96	56	10	46	0	0
GSE17156	Whole blood		Rhinovirus	GPL571	113	0	0	0	113
GSE20346	Whole blood	Adult	Bacteria, virus	GPL6947	81	36	0	0	19
GSE22098	Whole blood	Adult	Mycobacterium tuberculosis	GPL6947	274	81	52	0	0
GSE23140	Whole blood		Streptococcus pneumoniae	GPL6254	8	4	4	0	0
GSE25504	Whole blood		E.coli, virus, Group Strep,	GPL13667	20	6	6	4	3
GSE25504	Whole blood	Adult	Enterovirus	GPL570	5	3	1	0	0
GSE25504	Whole blood		E. coli, coag neg staph, E faecium	GPL6947	63	35	20	4	1
GSE27131	Whole blood	Adult	H1N1	GPL6244	21	7	0	0	14
GSE28750	Whole blood	Adult		GPL570	41	31	0	0	0
GSE29161	Whole blood		Endocarditis	GPL6480	15	10	5	0	0
GSE33341	Whole blood	Adult	E. coli, S. aureus	GPL571	94	43	32	19	0
GSE34205	Whole blood	Children	RSV	GPL570	101	22	0	0	79
GSE38246	Whole blood		DENV	GPL15615	113	8	0	0	105
GSE38900	Whole blood	Children	RSV	GPL10558	36	8	0	0	28
GSE40012	Whole blood	Adult	Influenza A	GPL6947	190	76	0	0	39
GSE40396	Whole blood	Children	Adenovirus, HHV6, Rhinovirus, E. coli, MRSA	GPL10558	65	22	4	3	35
GSE42026	Whole blood	Children	Gram positive bacteria, H1N1,RSV	GPL6947	92	33	18	0	41
GSE42834	Whole blood			GPL10558	356	143	0	0	0
GSE51808	Whole blood		DENV	GPL13158	56	9	0	0	28
GSE57065	Whole blood	Adult		GPL570	107	25	0	0	0
GSE60244	Whole blood	Adult	Bacteria, virus	GPL10558	158	40	0	0	71
GSE63990	Whole blood	Adult	Bacteria, virus	GPL571	280	90	0	0	117
GSE66099	Whole blood	Children		GPL570	276	47	0	199	30
GSE68310	Whole blood	Adult	Influenza A	GPL10558	880	0	0	0	725
GSE69528	Whole blood	Adult	B. pseudomallei	GPL10558	138	55	17	66	0
GSE69606	Whole blood			GPL570	43	17	0	0	26
GSE111368	Whole blood	Adult	H1N1, H3N2, influenza	GPL10558	359	130	0	0	229

**Validation set**									

GSE21802	Whole blood	Adult	H1N1	GPL6102	40	4	0	0	36
GSE13015	Whole blood	Adult	B. pseudomallei, E. coli	GPL6106	67	12	0	24	0
GSE40586	Whole blood		Spn	GPL6244	39	18	14	0	0
GSE103842	Whole blood	Children	RSV	GPL10558	74	12	0	0	62
GSE61821	Whole blood	Adult	H1N1	GPL10558	402	0	0	0	147
GSE9692	Whole blood	Children	Neisseria, Group B Strep, Group A Strep, Adenovirus	GPL570	45	29	8	2	6

**RNA-seq validation set**									

GSE157103	Whole blood	Adult	COVID-19	GPL24676	126	26			100
GSE176079	Whole blood		Dengue	GPL21290	27	9			18

Then, we separated them into a discovery set containing 3,409 samples from 34 cohorts and a validation set containing 374 samples from 6 cohorts. The discovery set was split into a training set and a test set (see STAR Methods). The training set included 817 non-infected, 229 Gram-positive bacteria-infected, 290 Gram-negative bacteria-infected, and 1,391 virus-infected samples, most of which were infected by RNA virus. The test set included 204 non-infected, 57 Gram-positive bacteria-infected, 73 Gram-negative bacteria-infected, and 348 virus-infected samples. The validation set included 75 non-infected, 22 Gram-positive bacteria-infected, 26 Gram-negative bacteria-infected, and 251 virus-infected samples (Figure 2D).

iPAGE was used to remove batch effect and integrate the host infection transcriptome cohorts (see STAR Methods). The result of Principal Component Analysis (PCA) demonstrated that the samples from the same cohorts tend to cluster together, whereas they are scattered around the space after iPAGE transformation (Figures 2B and 2C), indicating the decent performance of data integration.

### Multi-infectious detection model bvnGPS2 based on DGPs

After integrating training cohorts using iPAGE, we extracted gene pairs within an identical pathway, as genes involved in the same pathway are generally assumed to be functionally related. Based on Molecular Signatures Database (MsigDB), 186 gene sets derived from the curated gene sets (C2)-KEGG were separately used for gene pair selection. Then, the discriminant gene pairs (DGPs) for each infection type were selected as potential gene pair signatures (adjusted *p*-value< $10^{-16}$; Fisher’s Exact Test). Subsequentially, 7,955, 28,695, 30,422, and 15,961 infection-category-specific gene pairs were obtained for Gram-positive bacterial-infected, Gram-negative bacterial-infected, viral-infected, and non-infected groups, respectively.

**Figure 3:**
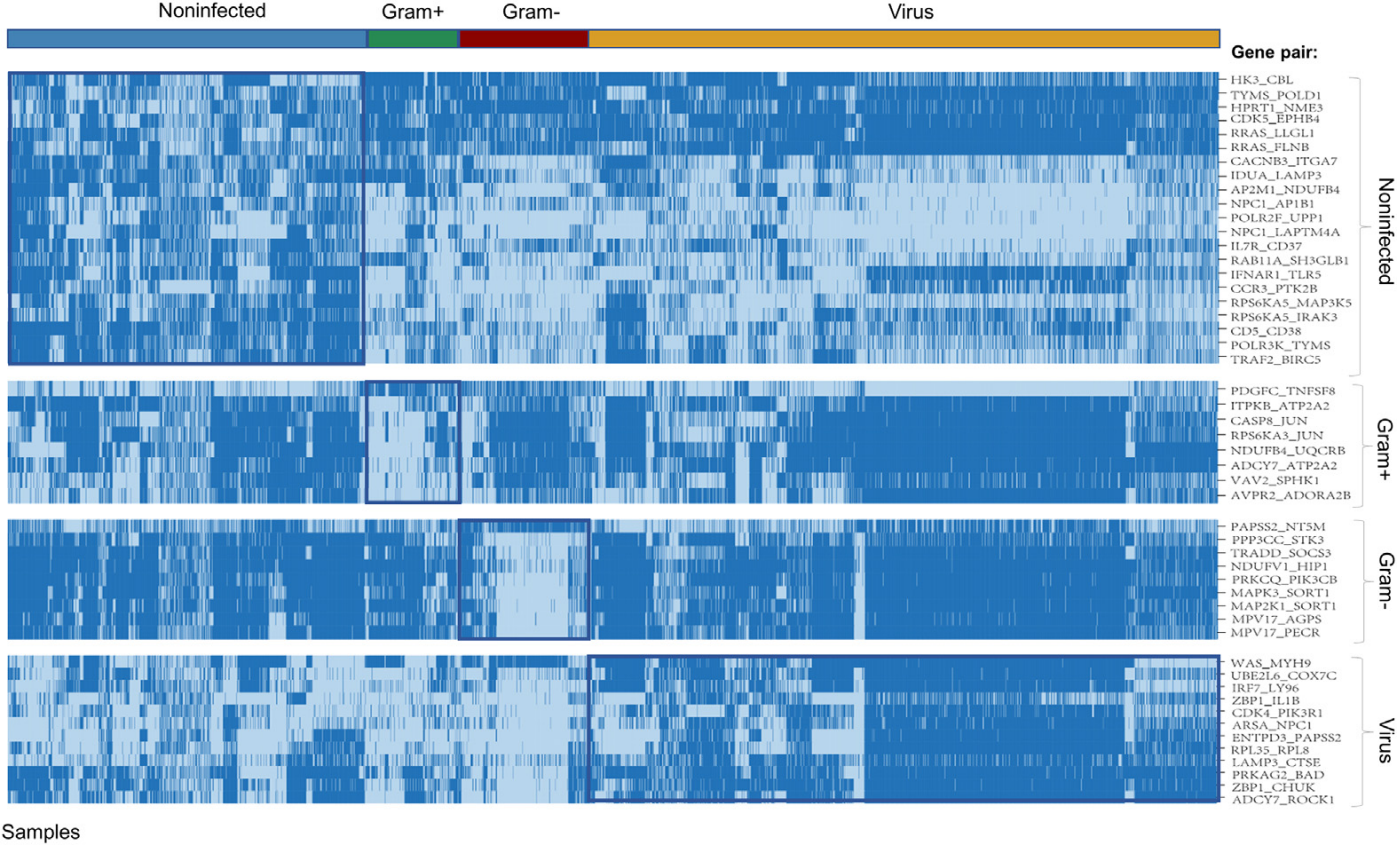
The heatmap of DGPs for the four infection categories Infection-specific gene pairs were highlighted in the blue frame. The blue color represents the first gene expressing higher than the second one in a DGP, while the gray color represents the first gene expressing lower than the second one.

Next, we performed LASSO and RF to identify the DGPs based on the feature importance according to the weights of each gene pair. Intersecting of the two DGPs lists from LASSO and RF, ultimately, we identified 21, 8, 9, and 12 DGPs respectively for non-infected, Gram-positive bacteria-infected, Gram-negative bacteria-infected, and virus-infected groups, respectively (Figure S2). The position of these DGPs in the chromosome are shown in the circos plots (Figures 2E–2H). Intuitively, the DGPs expressed distinctly between the four infection categories (Figure 3).

Finally, we merged the 50 DGPs as the final features and fed them into a deep neural network, which was constructed by two attention layers and five fully connected layers. With the attention mechanism, the network is able to pay more attention to those essential DGPs sample by sample (see STAR Methods). To account for the imbalance in sample sizes of the four groups, we adjusted the weights of each class during the network training. Ultimately, we developed bvnGPS2, the final decision model with attention mechanism, to detect host infections based on blood transcriptomic profiles.

### Performance evaluation

**Figure 4:**
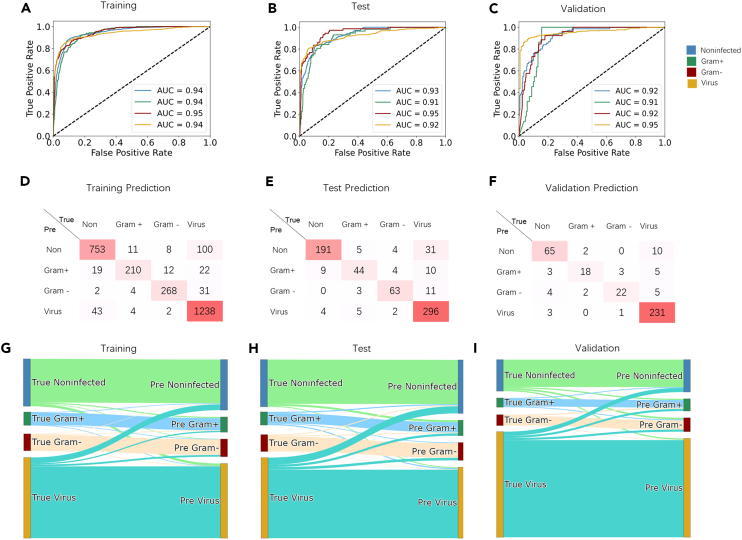
The infections predicted by bvnGPS2 in the training set, test set, and validation set (A–C) The ROC curves for the four infection categories in the training set, test set, and validation set. (D–F) Heatmap representation of bvnGPS2 performance. Each number represents the number of infected patients predicted by bvnGPS2. (G–I) Sankey diagram illustrating the number of true infection category and predicted infection category. True (left): true infection category, Pre (right): predicted category.

**Figure 5:**
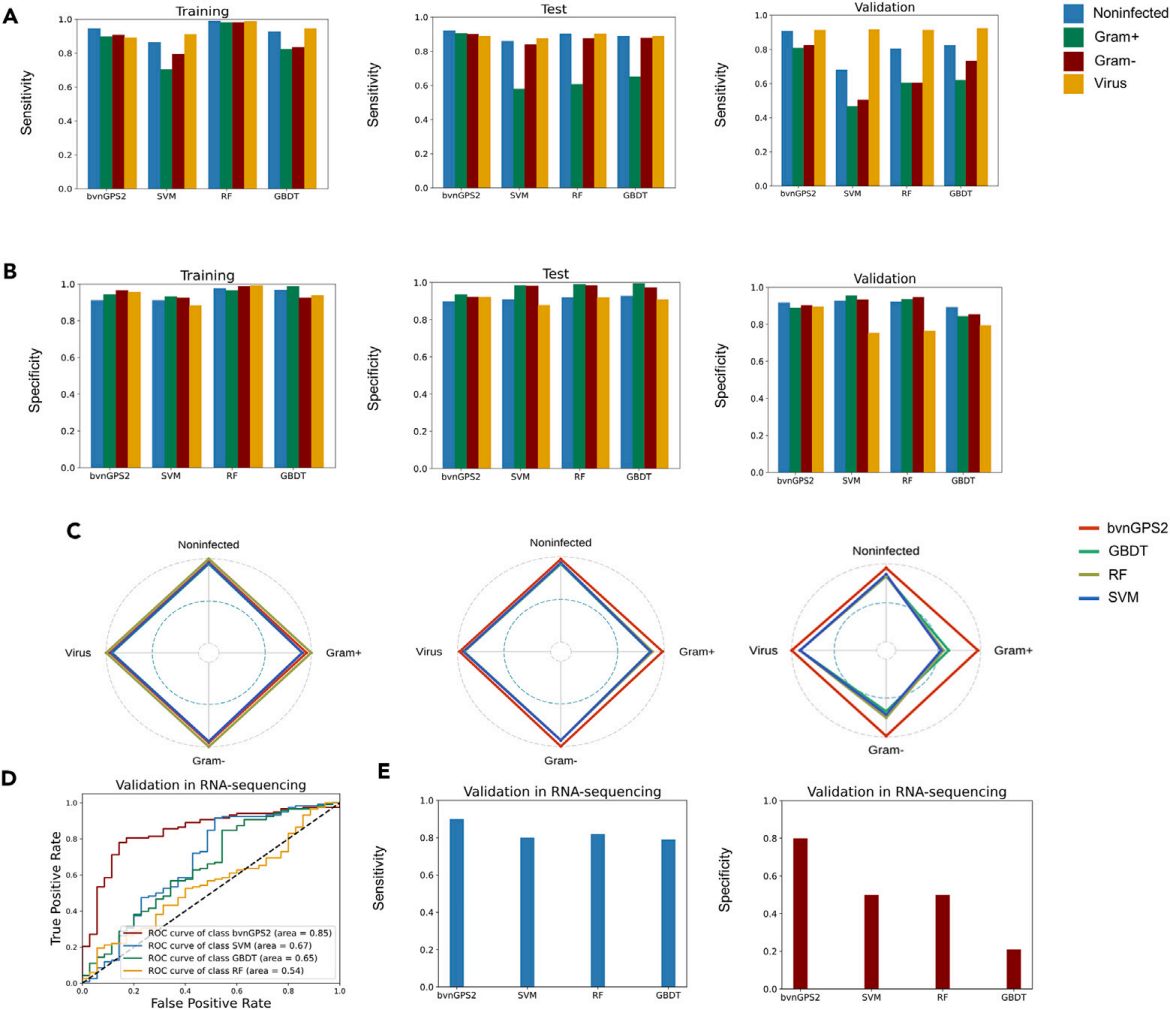
Comparison of bvnGPS2 to the commonly used machine learning methods (A) The sensitivity of the four methods for each infection category in the training set, test set, and validation set. (B) The specificity of the four methods for each infection category in the training set, test set, and validation set. (C) The radar plot shows the area under ROC curve of bvnGPS2 and the machine learning methods in four infection categories. (D) The ROC curves of bvnGPS2, SVM, GBDT, and RF in the RNA-seq validation set. (E) The sensitivities and specificities of bvnGPS2, SVM, GBDT, and RF in the RNA-seq validation set. SVM, Support Vector Machine; RF, Random Forest; GBDT, Gradient Boosting Decision Tree.

We comprehensively evaluated the performance of bvnGPS2 in the training set, the test set, and the validation set. In the training set, bvnGPS2 demonstrated the AUCs of 0.958 (95% CI, 0.932 to 0.971) for non-infection, 0.949 (95% CI, 0.917 to 0.962) for Gram-positive bacterial infection, 0.953 (95% CI, 0.936 to 0.974) for Gram-negative bacterial infection, and 0.960 (95% CI, 0.943 to 0.981) for viral infection (Figure 4A, Table 2). In the test set, bvnGPS2 achieved the AUCs of 0.939 (95% CI, 0.923 to 0.954), 0.948 (95% CI, 0.926 to 0.970), 0.965 (95% CI, 0.944 to 0.990), and 0.943 (95% CI, 0.925 to 0.960) for the four categories respectively (Figure 4B, Table 2). It demonstrated a good performance in the validation set with noninfected-vs.-others AUC of 0.915 (95% CI, 0.887 to 0.933), Gram-positive- vs.-others AUC of 0.936 (95% CI, 0.921 to 0.942), Gram-negative- vs.-others AUC of 0.954 (95% CI, 0.944 to 0.969), and virus- vs.-others AUC of 0.966 (95% CI, 0.955 to 0.972) (Figure 4C, Table 2). To evaluate the cross-platform performance of the developed model, we also included two RNA-seq cohorts for external validation (Table 1). In the integrated dataset (GSE157103 and GSE176079) based on Next-generation sequencing (NGS) platform, bvnGPS2 achieved an AUC of 0.853 (95% CI, 0.850 to 0.867) for virus-infection (Figure 5D, Table 2).

**Table 2:** Performance of bvnGPS2 and the common-used machine learning models

Infection Type and Metric	bvnGPS2			SVM			Random Forest			GBDT		
	Train	Test	Val	Train	Test	Val	Train	Test	Val	Train	Test	Val
Noninfected AUC	0.958	0.939	0.915	0.952	0.886	0.855	0.933	0.923	0.886	0.92	0.896	0.909
Gram+ AUC	0.949	0.948	0.936	0.906	0.815	0.509	0.9	0.903	0.815	0.949	0.932	0.896
Gram- AUC	0.953	0.965	0.954	0.939	0.89	0.661	0.905	0.89	0.817	0.962	0.93	0.92
Virus AUC	0.96	0.943	0.966	0.943	0.907	0.883	0.887	0.872	0.908	0.953	0.931	0.925
Noninfected Accuracy	0.925	0.905	0.905	0.948	0.896	0.897	0.972	0.901	0.885	0.944	0.903	0.903
Gram+ Accuracy	0.942	0.924	0.891	0.945	0.944	0.916	0.961	0.955	0.915	0.966	0.936	0.921
Gram- Accuracy	0.953	0.926	0.917	0.94	0.954	0.901	0.971	0.956	0.925	0.972	0.945	0.898
Virus Accuracy	0.919	0.902	0.913	0.946	0.897	0.906	0.989	0.898	0.873	0.932	0.882	0.917
Noninfected Sensitivity	0.933	0.923	0.886	0.928	0.853	0.786	0.958	0.872	0.73	0.91	0.887	0.838
Gram+ Sensitivity	0.9	0.903	0.815	0.81	0.632	0.365	0.977	0.651	0.64	0.803	0.648	0.578
Gram- Sensitivity	0.905	0.89	0.817	0.859	0.828	0.384	0.967	0.835	0.601	0.865	0.808	0.701
Virus Sensitivity	0.887	0.872	0.908	0.95	0.893	0.932	0.985	0.877	0.93	0.938	0.876	0.948
Noninfected Specificity	0.92	0.896	0.909	0.955	0.92	0.923	0.977	0.914	0.924	0.959	0.91	0.92
Gram+ Specificity	0.949	0.932	0.896	0.961	0.977	0.951	0.96	0.987	0.932	0.987	0.974	0.942
Gram- Specificity	0.962	0.93	0.92	0.957	0.972	0.936	0.973	0.97	0.945	0.986	0.967	0.913
Virus Specificity	0.953	0.931	0.925	0.943	0.901	0.853	0.991	0.911	0.764	0.929	0.895	0.853

Moreover, bvnGPS2 achieved high sensitivities and specificities in the four infection groups. The sensitivities of bvnGPS2 in the training set were 0.933, 0.900, 0.905, and 0.887, and the corresponding specificities were 0.920, 0.949, 0.962, and 0.953 for the non-infection, Gram-positive infection, Gram-negative infection, and viral infection (Figure 5A, Table 2). In the test set, sensitivities of bvnGPS2 were 0.923, 0.903, 0.890, and 0.872, while the specificities were 0.896, 0.932, 0.930, and 0.931 for the four infection categories (Figure 5B, Table 2). In the independent validation set, the sensitivities of bvnGPS2 were 0.886, 0.815, 0.817, and 0.908, while the specificities were 0.909, 0.896, 0.920, and 0.925, respectively (Figure 5C, Table 2). In the RNA-seq validation set, the sensitivity was 0.901 and the specificity was 0.802 for discriminating virus infection from non-infection (Figure 5E, Table 2).

Besides, we compared the performance of bvnGPS2 to the commonly used machine learning models, including support vector machine (SVM), random forest (RF), and gradient boosting decision tree (GBDT) concerning AUC, sensitivity, and specificity (Figure 5). bvnGPS2 demonstrated a higher sensitivity on non-infection, Gram-positive bacterial infection, and Gram-positive bacterial infection than the other models in the independent validation set (Figure 5A). Moreover, bvnGPS2 was more specific in identifying viral infection, while it achieved comparable performance in the other three types of infections (Figure 5B). Overall, the AUCs of bvnGPS2 for the four infections outperformed the other models in the test set and the validation set (Figure 5C). Therefore, bvnGPS2 with attention mechanism outperformed the commonly used machine learning models, indicating that bvnGPS2 is a robust and accurate indicator for different infection types.

### Biological functions of the DGPs

**Figure 6:**
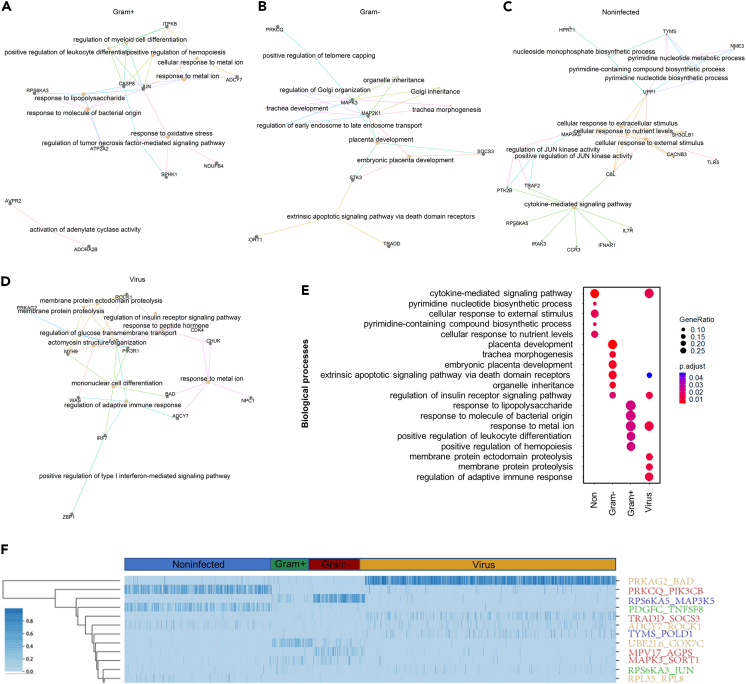
Functional analysis of discriminant gene pairs (DGPs) (A–D) The top twelve significant biological process involving the DGPs of Gram+ bacterial (A), Gram-bacterial (B), Noninfected (C), and Viral (D) infections in Gene Ontology. (E) DGPs of different infection categories are function-specific. (F) Heatmap of the top 12 DGPs with the highest attention weights of neural network with attention mechanism in each sample. The attention weights of DGPs are specifically different among different infection categories.

Biological functions of the DGPs in different infection types were separately characterized. The Gram+ DGPs are mainly involved in the response to molecule of bacterial origin, the positive regulation of leukocyte differentiation, and the response to oxidative stress (Figure 6A), which are key factors in bacterial infection.^50^ The Gram- DGPs included MAPK3 and MAP2K1 that enriched in functions related to immune response such as placenta development, trachea morphogenesis, and organelle inheritance (Figure 6B). The non-infection DGPs participated in the cytokine-mediated signaling pathway, pyrimidine nucleotide biosynthetic process, cellular response to external stimulus, cellular response to nutrient levels (Figure 6C). Although the viral DGPs were involved in some common biological pathways with the other three infection categories (Figures 6D and 6E), they are involved in some specific processes, including membrane protein proteolysis and regulation of adaptive immune response (Figure 6D), which has been reported to be associated with viral infection.^51^

## Discussion

Immune response gene expression has been widely used to identify diagnostic biomarkers in precision medicine, because of its effectiveness and robustness. Although previous studies detected infections taking advantage of the host gene expression, these studies may be weakened due to the small sample size, rendering them unreliable in clinical use.^2^^,^^10^^,^^12^ The model we built, however, is based on 40 published cohorts, including 4,949 samples, which is the largest dataset for training infection detection model to our knowledge. The large sample size improves the accuracy of our model, increasing the confidence and statistical interpretation of the results. In addition, bvnGPS2 is the first model to discriminate four infection categories, i.e., Gram-positive bacterial, Gram-negative bacterial, viral, and non-infections. These infection types covered in this study provide more valuable information in clinical settings and personalized treatments. Instead of differentially expressed genes, the DGPs used in this study are more convenient in removing batch effect and are comparable across distinct studies.

iPAGE simultaneously extracts the most generalizable information of multiple datasets as long as the quantities are monotonic, since it only concentrates on the relative expression (−1 or 1) of a pair of genes. The relative expressions of host response provided a simple but powerful way to overcome batch effect and integrate datasets from different resources in clinical practice. Assembling small dataset into big data to enlarge the sample size enables the application of complex deep learning models with more parameters, such as attention mechanism.

The attention mechanism could be quite suitable for gene expression, as it enriches different gene weights and includes global connectivity between gene pairs. In Figure 6F, we showed the top 12 gene pairs with the largest weights for all the samples. The gene pair *PRKAG2-BAD* has the largest weight in the viral infection, indicating the neural network pays more attention to *PRKAG2-BAD* when reviewing samples with the viral infection. Other gene pairs such as *TRADD-SOCS3, ADCY7-ROCK1*, and *TYMS-POLD1* may assist in the diagnosis of viral infection. Similarly, the neural network pays more attention to *MPV17-AGPS* and *MAPK3-SORT1* for the Gram-negative bacterial infected samples and *RPS6KA3-JUN* for the Gram-positive bacterial infections.

We applied iPAGE to separately identify the DGPs in each infection group, because using a multi-label network directly can cause the imbalanced weights between different infection groups. The attention mechanism, however, given the integrated DGPs from four infection groups, considered the interactions among different gene pairs from different infection groups, thus utilizing more information of the features to improve the classification performance.

In conclusion, we proposed a robust and concise model, bvnGPS2, for discriminating Gram-positive bacterial, Gram-negative bacterial, viral, and non-infections. By incorporating the iPAGE algorithm and attention mechanism, bvnGPS2 outperformed the traditional machine learning methods. Given its effectiveness and robustness, bvnGPS2 has the potential to be applied in assisting the diagnosis of infections in clinical practice. The framework of bvnGPS2 could be further generalized to develop multi-class diagnostic models for other diseases.

### Limitations of the study

There are also some limitations of this work. Notice that in this work most virus infection is caused by RNA viruses, although GSE40396 and GSE9692 contain samples infected by the DNA-virus, adenovirus. It may limit the developed model’s ability to identify DNA virus infection. This is because only a few gene expression profiles of DNA virus infection were found in the GEO database. Much more RNA virus datasets than DNA virus resulting in the imbalanced training may lead to a poor performance. As RNA viruses and DNA viruses induce distinct innate immune pathways upon infection and accordingly have different transcriptome signatures, it is also important to develop a model to discriminate RNA viruses and DNA viruses, or a five-categories classifier that can discriminate non-infection, Gram-positive bacteria, Gram-negative bacteria, RNA viruses, and DNA virus infections. We would like to leave this as a future work when sufficient DNA virus infection data are available. Besides, the sample sizes of Gram-positive and Gram-negative infection are relatively small, which may limit the robustness and generalizability of this model.

Of note, although the current model developed from micro-array data performed well on the two RNA-seq virus-infection cohorts, it would be essential to incorporate a broader range of RNA-seq data for comprehensive validation when more RNA-seq cohorts are available. Here, we appeal for more RNA-seq cohorts in infections, especially in DNA-viral infection.

## STAR★Methods

### Key resources table

**Table:** 

REAGENT or RESOURCE	SOURCE	IDENTIFIER
**Deposited data**		

Code for processing data and building the model.	This paper	https://zenodo.org/doi/10.5281/zenodo.10896227

**Software and algorithms**		

Python 3.9	The Python Software Foundation	https://www.python.org/
R 4.3.2	R Core Team	https://www.R-project.org/

### Resource availability

#### Lead contact

Further information and requests for resources and reagents should be directed to and will be fulfilled by the lead contact, Lixin Cheng (easonlcheng@gmail.com).

#### Materials availability

This study did not generate new materials.

#### Data and code availability

• Data used for training and testing are available in Table 1. All data are public and available in GEO, and can also be obtained from the lead contact.
• The R script for data downloading in the manuscript and the code conducted on Python 3.9 are available on https://zenodo.org/doi/10.5281/zenodo.10896227.
• Any additional information required to reanalyze the data reported in this paper is available from the lead contact upon request.

### Method details

#### Data preparation

To collect sufficient data, we performed a comprehensive search in the Gene Expression Omnibus (GEO) database and ultimately selected 40 cohorts across ten countries (Table 1). Then we used GEOquery (2.70.0) and AnnoProbe (0.1.0) to download and label the data, resulting in 4,949 samples infected by Gram-positive bacteria, Gram-negative bacteria, or RNA viruses.^29^ Samples with unknown infection, multiple infection records, multiple pathogens or non-infection but other diseases were eliminated. For instance, as samples in GSE61821 were sequentially profiled on the first day and the 28^th^ day, the latter was excluded from our study. Finally, 3,783 samples were used for subsequent analysis, including 308 Gram-positive bacterial infected samples, 389 Gram-negative bacterial infected samples, 1,990 virus-infected samples, and 1,096 non-infected samples. The patients infected with Gram-positive bacteria mainly covered S.pneumoniae, S.aureus, and E faecium, while the Gram-negative bacteria mainly included B.pseudomallei, and E.coli. The virus group mainly focused on RNA virus such as H1N1, RSV, and Rhinovirus, although GSE40396 and GSE9692 contain samples infected by the DNA-virus, Adenovirus. We identified 4,164 common genes among different cohorts using Numpy (1.24.3) and Pandas (2.1.1) in Python (3.9).

We integrated 34 out of 40 cohorts as the discovery set and randomly split them into a training set (80%; N=2,727) for feature selection, model training, and parameter optimization, and a test set (20%; N=682) for internal evaluation (Figure 1A). The remaining six cohorts, GSE21802, GSE13015, GSE40586, GSE103842, GSE61821, and GSE9692, were integrated as an independent validation set (N=374) for external evaluation. We also integrated two RNA-sequencing cohorts GSE157103 and GSE176079 to test our model’s performance in RNA-sequencing data. The GSE157103 contains 100 COVID-19 samples and 26 healthy samples, the GSE176079 contains 9 healthy samples and 18 samples with Dengue.

#### Individualized pairwise analysis of gene expression

To improve the robustness and reliability of the model,^11^^,^^27^^,^^28^^,^^30^ we discovered consistent gene features based on relative expression between two genes within a sample using our previously developed algorithm, individualized Pairwise Analysis of Gene Expression (iPAGE).^11^^,^^12^^,^^21^^,^^22^^,^^25^^,^^31^^,^^32^^,^^33^ Generally, the absolute expression intensity of genes was affected by the batch effect, measuring platforms, and the amplification reagent used.^34^ According to the previous studies, the relative expression between two genes within a sample is more robust and reliable.^11^^,^^27^^,^^28^^,^^30^ Therefore, we used the relative expression as a base to select consistent gene features.

We used the gene sets from the Molecular Signatures Database (MsigDB)^35^ to stratify the detected genes into 186 pathways and then separately performed gene pair selection. iPAGE first computed the relative expression of all gene pairs in each pathway for each sample in parallel. Then it filtered out the gene pairs that showed significant differences across samples. We used $h_{i}^{k}$ to represent the raw expression intensity of the *i*-th gene for sample *k*. Let $r_{i,j}^{k}$ denote the relative expression of gene *i* and gene *j* within the sample *k*. If $h_{i}^{k}$ > $h_{j}^{k}$, then $r_{i,j}^{k}$ is 1; otherwise $r_{i,j}^{k}$ is −1. Using $Y^{k}=1$ to represent positive and $Y^{k}=0$ to represent negative, the contingency table can be calculated as follow.

**Table:** 

	$h_{i}^{k}>h_{j}^{k}$	$h_{i}^{k}\leq h_{j}^{k}$
Positive	$\frac{1}{2}\sum_{k=1}^{n}\left(r_{i,j}^{k}+1\right)\times Y^{k}$	$\frac{1}{2}\sum_{k=1}^{n}{(1-r}_{i,j}^{k})\times Y^{k}$
Negative	$\frac{1}{2}\sum_{k=1}^{n}\left(r_{i,j}^{k}+1\right)\times {(1-Y}^{k})$	$\frac{1}{2}\sum_{k=1}^{n}{(1-r}_{i,j}^{k})\times {(1-Y}^{k})$

Then, we used Fisher’s Exact Test with Bonferroni corrected false discovery rate as 1e-16 to screen the gene pairs with significantly different distributions between the interested and other samples (one vs. others).

#### Identification of discriminant gene pairs

Least absolute shrinkage and selection operator (LASSO) is a regression algorithm with normalization designed for the situation when the number of features far exceeds the number of samples.^36^ The normalization penalizes large model and selects the minimum features considered as valuable features. In this study, LASSO further refined gene pairs obtained from iPAGE. The loss function of LASSO is defined as

(Equation 1) $$R=\frac{1}{2N}\times \|y-Xw{\|}_{2}^{2}+\alpha *\|w{\|}_{1}$$

were set to select the least number of discriminant gene pairs. As a result, 14, 40, 64, and 57 gene pairs were identified for Gram-positive infection, Gram-negative infection, virus infection, and non-infection, respectively.

In addition, Random Forest (RF) has been widely used as classifiers in machine learning, especially when the number of variables is larger than the number of observations.^37^ RF dissects the complex interaction between variables and returns the variable importance by aggregating many decision trees and judging with majority voting.^38^ We applied RF to prioritize the gene pairs in each type of infection and select the most discriminant gene pairs. The number of estimators was set as 108. We selected the gene pairs with higher importance (higher than 0.001) and intersected them with gene pairs obtained through LASSO. We named the selected gene pairs as discriminant gene pairs (DGPs). All these steps were conducted in the Python environment with Scikit-learn (1.3).^39^

#### Decision model based on the attention mechanism

The attention mechanism is an effective deep learning framework in natural language processing and computer vision.^40^^,^^41^^,^^42^^,^^43^^,^^44^ It can adaptively adjust the attention to different features regarding samples and extract the global connectivity between features. In this work, we built a seven-layer deep neural network, bvnGPS2 (Figure 1B), which included two attention layers to identify different infection types based on the gene pairs selected by LASSO and RF. The model input is the training dataset constructed from the identified 50 gene pairs, with a shape of (2727, 50), where each sample contained fifty features. For each feature, i.e. (gene1, gene2), we assigned a value 1 if gene1 > gene2, or -1 otherwise.

We applied two attention layers with RELU and Softmax as the first and the second layers’ activation function.^45^ Five dense layers with RELU as activation function were stacked afterwards. The final layer used Softmax to predict the probabilities of the four types of infections. AdamW was applied as the optimizer and the learning rate was set as 0.001.^46^ The model contains 6,252 trainable parameters in total. 80% of the discovery data were used to train the model, and 20% were used to optimize parameters with early stopping, which monitors the loss in the test set to determine the termination of the training process.

#### Evaluation metrics

We evaluated the performance of bvnGPS2 using Area Under receiver operating characteristic Curve (AUC) in noninfected-vs.-others, Gram-positive-vs.-others, Gram-negative-vs.-others, and virus-vs.-others groups respectively.^47^ Besides, sensitivity, specificity, and accuracy were calculated for the training set, test set, and validation set as follows:

(Equation 2) $$Sensitivity=\frac{TP}{TP+FN}$$

(Equation 3) $$Specificity=\frac{TN}{TN+FP}$$

(Equation 4) $$Accuracy=\frac{TP+TN}{TP+TN+FP+FN}$$

where TP represents the number of true positive samples, TN represents the number of true negative samples, FP represents the number of false positive samples, and FN represents the number of false negative samples. High sensitivity indicates the model identifying the infection category correctly, and high specificity refers to the model less likely to classify the negative samples as positive.

#### Functional analysis

We further demonstrated the functional characteristics of the DGPs using clusterProfiler (3.17),^48^ which uses hypergeometric distribution test to evaluate the statistical significance of function enrichment in the Gene Ontology (GO) terms.^49^

### Quantification and statistical analysis

The iPAGE and Lasso analysis for the training set (n=2,727) are conducted using Numpy (1.24.3) and Pandas (2.1.1) in Python (3.9). For the Fisher’s Exact Test in iPAGE, we set the Bonferroni corrected false discovery rate as 1e-16. And for Lasso analysis, we set α=0.05 and β=1. For the functional characteristics of the DGPs, we used clusterProfiler (3.17) in R (4.3).

For the data inclusion and exclusion, we eliminated the samples with unknown infection, multiple infection records, multiple pathogens or non-infection but other diseases in the original dataset. The detailed amounts and criteria are exhibited in Figure S1. Moreover, the training set and test set are split from the discovery set with a ratio as 4:1, and we implemented this process by the Scikit-learn (1.3) package in Python with the random state as 1. All of these descriptions can also be found in the Method details section.
