# Supplementary material for: Deep learning model to discriminate diverse infection types based on pairwise analysis of host gene expression
Source: iScience. 2024 May 7;27(6):109908. doi: 10.1016/j.isci.2024.109908 (PMC11141160; doi:10.1016/j.isci.2024.109908)
Supplement: Document S1. Figures S1–S3 [file mmc1.pdf]

iScience, Volume *27*

Supplemental information

Deep learning model to discriminate diverse infection types based on pairwise analysis of host gene expression

Jize Xie, Xubin Zheng, Jianlong Yan, Qizhi Li, Nana Jin, Shuojia Wang, Pengfei Zhao, Shuai Li, Wanfu Ding, Lixin Cheng, and Qingshan Geng

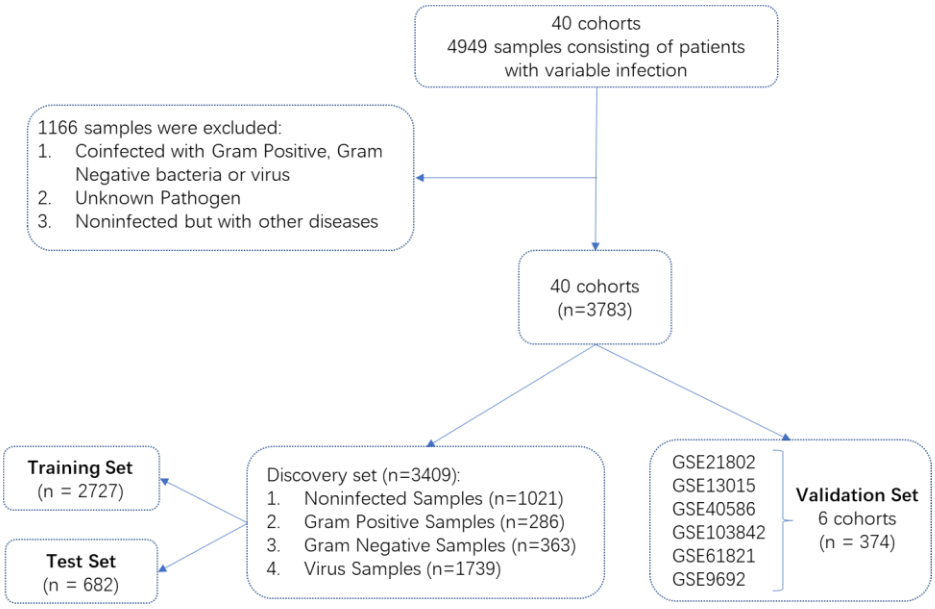

Figure S1. Inclusion and exclusion criteria of datasets. Related to Figure 2.

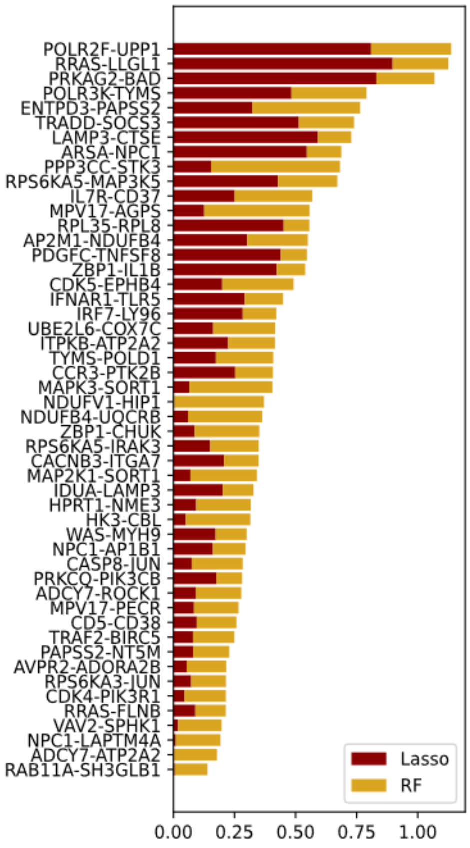

Figure S2. The infection-category-specific gene pairs (ICSPs) identified using LASSO and Random Forest. Related to Figure 2.

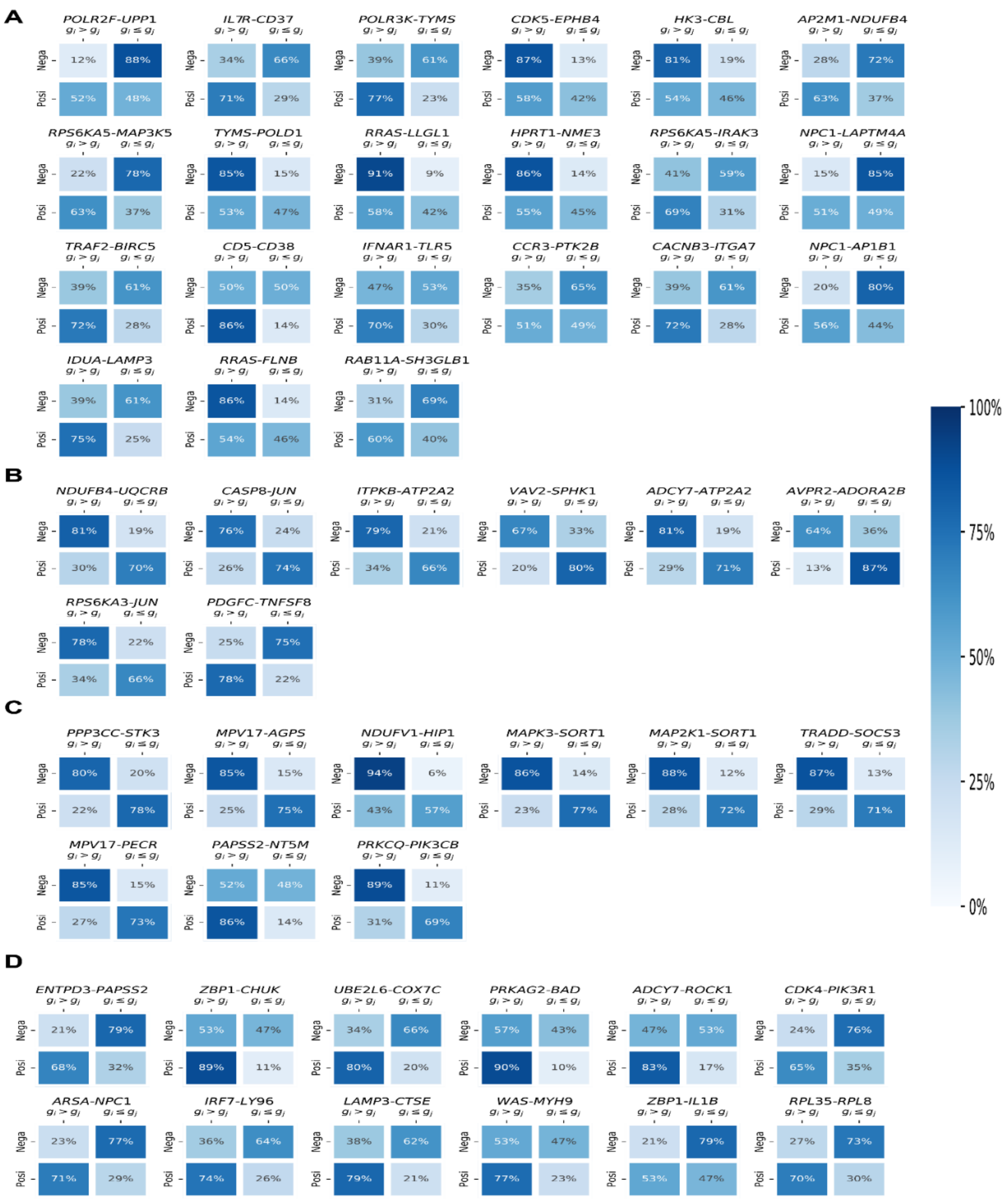

Figure S3. Contingency table of the determined ICSPs for non-infection (A), gram+ (B), gram- (C), and viral infection (D), respectively. Related to Figure 3.
